# Distribution and prognostic value of high-sensitivity cardiac troponin T and I across glycemic status: a population-based study

**DOI:** 10.1186/s12933-023-02092-z

**Published:** 2024-02-24

**Authors:** Jiajun Zhang, Xiaoxing Li, Shenglin Zhang, Zhen Wang, Rui Tian, Feng Xu, Yuguo Chen, Chuanbao Li

**Affiliations:** 1 Department of Emergency Medicine, Qilu Hospital of Shandong University, 107 Wenhua Xi Road, Jinan, Shandong 250012 People’s Republic of China; 2 Shandong Provincial Clinical Research Center for Emergency and Critical Care Medicine, Institute of Emergency and Critical Care Medicine of Shandong University, Chest Pain Center, Qilu Hospital of Shandong University, Jinan, China; 3 Key Laboratory of Emergency and Critical Care Medicine of Shandong Province, Key Laboratory of Cardiopulmonary-Cerebral Resuscitation Research of Shandong Province, Shandong Provincial Engineering Laboratory for Emergency and Critical Care Medicine, Qilu Hospital of Shandong University, Jinan, China; 4 Shandong Key Laboratory: Magnetic Field-Free Medicine & Functional Imaging (MF), Qilu Hospital of Shandong University, Jinan, China; 5 NMPA Key Laboratory for Clinical Research and Evaluation of Innovative Drug, Qilu Hospital of Shandong University, Jinan, China; 6 Department of Geriatrics, Qilu Hospital, Shandong University, Jinan, Shandong China

**Keywords:** Diabetes, Prediabetes, High-sensitivity, Cardiac troponin, Risk stratification

## Abstract

**Background:**

Whether distributions and prognostic values of high-sensitivity cardiac troponin (hs-cTn) T and I are different across normoglycemic, prediabetic, and diabetic populations is unknown.

**Methods:**

10127 adult participants from the National Health and Nutrition Examination Survey 1999–2004 with determined glycemic status and measurement of at least one of hs-cTn assays were included, from whom healthy participants and presumably healthy diabetic and prediabetic participants were selected to investigate pure impacts of glycemic status on distributions of hs-cTn. The nonparametric method and bootstrapping were used to derive the 99th upper reference limits of hs-cTn and 95% CI. Participants with available follow-up and hs-cTn concentrations of all 4 assays were included in prognostic analyses. Associations of hs-cTn with all-cause and cardiac-specific mortality were modeled by Cox proportional hazard regression under the complex survey design. The incremental value of hs-cTn to an established risk score in predicting cardiac-specific mortality was assessed by the 10-year area under time-dependent receiver operating characteristic curve (AUC) using the Fine-Grey competing risk model.

**Results:**

Among 9714 participants included in prognostic analyses, 5946 (61.2%) were normoglycemic, 2172 (22.4%) prediabetic, and 1596 (16.4%) diabetic. Hyperglycemic populations were older than the normoglycemic population but sex and race/ethnicity were similar. During the median follow-up of 16.8 years, hs-cTnT and hs-cTnI were independently associated with all-cause and cardiac-specific mortality across glycemic status. In the diabetic population, adjusted hazard ratios per 1-standard deviation increase of log-transformed hs-cTnT and hs-cTnI (Abbott) concentrations were 1.77 (95% CI 1.48–2.12; P < .001) and 1.83 (95% CI 1.33–2.53; P < .001), respectively, regarding cardiac-specific mortality. In the diabetic but not the normoglycemic population, adding either hs-cTnT (difference in AUC: 0.062; 95% CI 0.038–0.086; *P* < 0.001) or hs-cTnI (Abbott) (difference in AUC: 0.071; 95% CI 0.046–0.097; *P* < 0.001) would significantly increase the discriminative ability of the risk score; AUC of the score combined with hs-cTnT would be further improved by incorporating hs-cTnI (0.018; 95%CI 0.006–0.029; *P* = 0.002). The 99th percentile of hs-cTnT of the presumably healthy diabetic population was higher than the healthy population and had no overlap in 95% CIs, however, for hs-cTnI 99th percentiles of the two populations were very close and 95% CIs extensively overlapped.

**Conclusions:**

Hs-cTnT and hs-cTnI demonstrated consistent prognostic associations across glycemic status but incremental predictive values in hyperglycemic populations only. The susceptibility of hs-cTnT 99th percentiles to diabetes plus the additive value of hs-cTnI to hs-cTnT in diabetic cardiovascular risk stratification suggested hs-cTnI and hs-cTnT may be differentially associated with glycemic status, but further research is needed to illustrate the interaction between hyperglycemia and hs-cTn.

**Supplementary Information:**

The online version contains supplementary material available at 10.1186/s12933-023-02092-z.

## Background

Cardiac troponin (cTn) has been widely used to rule in and rule out acute myocardial infarction [1]. In the era of high sensitivity, cTn could be detected at a very low concentration, facilitating early diagnosis of myocardial infarction [1]. A solid body of evidence has demonstrated that elevated levels of serum high-sensitivity cTn (hs-cTn) provided significant prognostic information even though lower than the upper reference limit (URL), serving as a powerful biomarker of myocardial injury in cardiovascular risk stratification besides differential diagnosis of acute chest pain [2, 3].

Despite the excellent correlation between hs-cTnT and hs-cTnI in the context of acute coronary syndrome [1, 4], in direct comparisons of hs-cTnT vs. hs-cTnI under stable medical conditions, hs-cTnI and T were only modestly correlated [5, 6]. Stable conditions such as neuromuscular disease or stable coronary artery disease may be differentially associated with hs-cTnT and hs-cTnI whereby elevated hs-cTnT and hs-cTnI were presumed to represent distinct pathophysiologic processes [5–10].

Hyperglycemia may also contribute to chronic myocardial injury via oxidative stress, inflammation, etc., releasing a trace level of cTn even with no evident atherosclerotic cardiovascular disease (CVD) [11, 12]. Some preliminary studies found hs-cTnT was more strongly associated with diabetes than hs-cTnI suggesting hyperglycemia may also differentially influence hs-cTnT and hs-cTnI, but at present little is known about this [5–7].

Accordingly, the objective of the present study is twofold, first to investigate distributions and prognostic associations of hs-cTnT and hs-cTnI across glycemic status, second to illustrate the differential interaction of chronic hyperglycemia with hs-cTnT and hs-cTnI by contrasting characteristics of hs-cTnI and hs-cTnT in hyperglycemic populations to that in the nonglycemic population.

## Method

### Study population

The present study was based on data obtained from the National Health and Nutrition Examination Survey (NHANES) 1999–2004 cycles. The NHANES is a population-based program of surveys administered by the National Center for Health Statistics to monitor the health and nutritional status of civilian, non-institutionalized adults and children across the United States. Participants were recruited from a nationally representative sample of the US population via a complex, stratified, multistage probability cluster sampling design. Cross-sectional socio-demographic, dietary, and laboratory data and personal history were acquired by structured in-person interviews, uniform physical examination, and standardized laboratory testing. More descriptions of the methodology in data collection were detailed on the official website of NHANES [13]. The National Center for Health Statistics institutional review board approved the program protocol. Written informed consent was obtained from all participants. All data presented in this study is publicly available and can be freely downloaded from the official website of NHANES (https://wwwn.cdc.gov/nchs/nhanes/default.aspx). The present study was approved by the Institutional Review Board at our institution and was exempted from ethical oversight because de-identified data in the publicly available database was used.

Data from NHANES is released in a 2-year cycle. Our analysis was based on NHANES survey cycles 1999–2000, 2001–2002, and 2003–2004 when concentrations of 4 hs-cTn assays were available. 10 127 adult participants with determined glycemic status and measurement of at least one of the hs-cTn assays were included in this study, from whom healthy populations of each hs-cTn assay were selected. 9725 participants had available hs-cTn concentrations of all 4 assays, among whom 9714 with available follow-up were included to investigate prognostic and predictive values of hs-cTnT and hs-cTnI across glycemic status.

### Hs-cTn measurement

Surplus serum specimens in the NHANES 1999–2004 cycles were tested for hs-cTnT (Roche), hs-cTnI (Abbott), hs-cTnI (Siemens) and hs-cTnI (Ortho). These samples had been stored at − 80 ℃ from 1999–2004, the majority of which were never thawed and re-frozen before measurement. Measurements of hs-cTn of 4 assays were performed at the University of Maryland School of Medicine during 2018–2020. Hs-cTnT was measured on the Roche Cobas e601 analyzer using the 5th generation Elecsys assay. Hs-cTnI (Abbott) was measured on ARCHITECT i2000SR analyzer. Hs-cTnI (Siemens) and hs-cTnI (Ortho) were measured using Centaur XPT and Vitros 3600, respectively.

Analytical characteristics (the lower limit of detection (LoD), the limit of quantitation, the manufacturer-proposed 99th percentile, and coefficients of variation at corresponding values) of hs-cTn assays have been illustrated by the study of McEvoy et al. and the International Federation of Clinical Chemistry Committee (IFCC) on Clinical Application of Cardiac Bio-Markers in detail [14, 15]. In brief, the LoDs are 3, 1.7, 1.6, and 0.39 ng/L for hs-cTnT (Roche), hs-cTnI (Abbott), hs-cTnI (Siemens), hs-cTnI (Ortho), respectively. The manufacturer-proposed overall (not sex-specific) 99th percentiles are 19, 28, 46.5, and 11 ng/L for hs-cTnT (Roche), hs-cTnI (Abbott), hs-cTnI (Siemens), and hs-cTnI (Ortho), respectively. More information on laboratory methodology and analytic notes can be found in the online help file on the official website of NHANES [16].

### Glycemic status

Participants who have a self-reported history of diabetes or currently taking glucose-lowering agents, or fasting plasma glucose ≥ 126 mg/dl (7.0 mmol/L), or HbA1c ≥ 6.5% (48 mmol/mol) were classified as diabetes [17, 18]. In participants not meet the criteria of diabetes, prediabetes was defined as fasting plasma glucose values of 100–125 mg/dL (5.6–6.9 mmol/L) or HbA1c values of 5.7–6.4% (39–47 mmol/mol) [17, 18]. People who meet definitions of neither diabetes nor prediabetes were regarded as normoglycemia.

### Clinical and laboratory covariables

Demographic characteristics (age, sex, race-ethnicity), smoking status, and medical history of rheumatoid arthritis, cancer, lung disease, liver disease, current thyroid disease, and CVD (congestive heart failure, coronary heart disease, angina pectoris, myocardial infarction, and stroke) were identified by in-person interview and questionnaires. Race-ethnicity was categorized as non-Hispanic White, non-Hispanic Black, Mexican American, and others. Participants who smoked every day or some days were classified as current smokers.

Participants’ height (m), weight (kg), and blood pressure (mmHg) were measured by trained physicians at the mobile examination center. According to body mass index (weight divided by the square of height), participants were categorized into underweight (< 18.0 kg/m^2^), normal (18–35.0 kg/m^2^), and obese (≥ 35.0 kg/m^2^). Hypertension was defined as having a self-reported history of the diagnosis or taking anti-hypertensive agents now, or the average systolic blood pressure ≥ 140 mmHg, or the average diastolic blood pressure ≥ 90 mmHg.

Laboratory assays to determine urinary creatinine and albumin, and serum or plasma levels of N-terminal pro-brain natriuretic peptide (NT-proBNP), Cystatin C, HbA1c, alanine transaminase, aspartate aminotransferase, albumin, gamma-glutamyl transferase, bilirubin, creatinine, urea nitrogen, glucose, cholesterol, triglycerides, uric acid, homocysteine, hemoglobin, platelet and white blood cell count, and C‐reactive protein were reported in detail elsewhere (Additional file 1) [13]. The estimated glomerular filtration rate (eGFR) was calculated by the new Chronic Kidney Disease Epidemiology Collaboration equation based on serum creatinine and cystatin C without race [19]. Albuminuria was defined as urine albumin to creatinine ratio (UACR) > 30 mg/g.

### Healthy reference

According to instructions from the IFCC Committee [20], the healthy reference population included adult participants without current pregnancy. Subjects were subsequently excluded if they had diabetes, prediabetes, self-reported history of cardiocerebrovascular diseases, NT-proBNP > 125 ng/L, chronic diseases that could affect the heart (cancer, rheumatoid arthritis, and lung, liver, and current thyroid disease), currently taking prescribed medications for hyperlipidemia or hypertension, overnight hospitalization in past 12 months, abnormal weight (underweight or obese), chronic renal disease (albuminuria or eGFR < 60 ml/min/1.73 m^2^). Current smokers were also excluded. To investigate the pure impact of glycemic status on the 99th percentile, diabetes and prediabetes who meet the same criteria as the healthy reference except glycemic requirements were selected to calculate the 99th percentile.

### Follow-up

The public-use-linked mortality follow-up through December 31, 2019, was acquired from the National Death Index. The number of months of follow-up started from the NHANES mobile examination center date and ended at the time of death or December 31, 2019. As provided by the National Death Index, the presence of the International Classification of Diseases–Tenth Revision codes I00–I09, I11, I13, I20–I51, and I60–I69 indicated a cardiac-specific death.

### Statistical analysis

All statistical analyses were conducted under the complex survey design of NHANES using appropriate survey weights to obtain estimates that could be generalized to the US population. Unweighted analyses would be specified if the survey design was not considered. Continuous variables were expressed as weighted means (standard error (SE)) or median (interquartile range), and categorical variables were expressed as weighted proportions (SE). Proportions of missing values of all covariables were < 5% so the values were imputed by the multiple chained imputations method*.*

The nonparametric method not considering survey weights was applied to derive the 99th percentiles of hs-cTn of four assays. As recommended by IFCC, outliners were excluded using Reed/Dixon criteria, and 95% confidence intervals (CIs) of percentiles were obtained via bootstrapping [15, 20]. Unweighted age- and sex-adjusted Spearman’s rank correlation coefficients between hs-cTn and clinical covariables and between hs-cTnI (Abbott) and hs-cTnT were calculated across glycemic status.

Cox proportional hazard regression was performed to investigate the association between the per 1-standard deviation increase of natural log-transformed hs-cTn concentrations of four assays and risk change of all-cause and cardiac-specific mortality across glycemic status. In multivariable analysis within each glycemic stratum, hs-cTn were adjusted in model 1 which incorporated age, sex, race-ethnicity, and other variables from the Pooled Cohort Equation (PCE) model (smoking status, systolic blood pressure, taking prescribed medication for hypertension, total cholesterol, and high-density lipoprotein cholesterol) [21]. In model 2, NT-proBNP and eGFR were additionally adjusted based on model 1.

To investigate incremental predictive values of hs-cTn (log-transformed), we compare the area under the time-dependent receiver operating characteristic curve (AUC) of the recalibrated PCE (rPCE) model vs. rPCE plus either hs-cTnT or hs-cTnI or both in predicting 10-year cardiac-specific mortality. Before comparisons, the baseline hazard of the PCE model within each glycemic stratum was recalibrated by fitting the Fine-Grey competing risk model with 10-year risk scores calculated from the original PCE model. In Fine-Grey competing risk models, non-cardiac death was treated as a competing event, and survey weights were not considered in these individual risk predictions. The predictive value of hs-cTnI was mainly focused on hs-cTnI (Abbott) as only hs-cTnI (Abbott) demonstrated consistent and robust prognostic value among the three hs-cTnI assays.

In sensitive analysis, we also investigated prognostic and predictive values of hs-cTn in the primary-prevention population who had no CVD history. A two-sided P value < 0.05 was considered significant. All statistical analysis was performed on R software (version 4.2.3).

## Result

### Distributions and 99th percentiles of hs-cTn

Among 9714 participants with full availability of four hs-cTn assays, 5946 (61.2%) were normoglycemic, 2172 (22.4%) prediabetic, and 1596 (16.4%) diabetic. Diabetic and prediabetic populations were older than the normoglycemic population. Prevalences of obesity, hypertension, and albuminuria in hyperglycemic populations were higher than those in the normoglycemic population (Additional file 2). However, the proportion of current smokers was slightly higher in the normoglycemic population.

Distributions of serum hs-cTn concentrations across glycemic status were presented in the violin plot (Additional file 3). For all hs-cTn assays, weighted median concentrations and weighed percentages of concentrations above the manufacturer-proposed URL or the LoD were smallest in the normoglycemic but highest in the diabetic population; prediabetes served as an intermediate status (Table 1). Proportions of hs-cTnT concentrations > the 99th URL and > the LoD were higher than hs-cTnI irrespective of glycemic status. Moreover, the absolute difference in the proportion of hs-cTnT concentrations above the manufacturer-proposed URL between the normoglycemic and the diabetic population (2.4% vs. 15.6%) was larger than that of hs-cTnI (0.5% vs. 2.4%, for the Abbott assay) but for the proportion of concentrations > the LoD, the absolute difference of hs-cTnT was smaller.

**Table 1:** Survey-weighted distributions of hs-cTn in the population with full availability of four hs-cTn assays

	Normoglycemia	Prediabetes	Diabetes
No. of pts before weighting	5946	2172	1596
Concentrations of hs-cTn, ng/L^a^			
hs-cTnT	4.4 (3.3–6.3)	6.1 (4.5–9.1)	8.8 (5.4–14.5)
hs-cTnI (Abbott)	1.4 (0.9–2.3)	2.1 (1.4–3.2)	2.7 (1.6–5.0)
hs-cTnI (Siemens)	2.1 (1.0–4.0)	3.1 (1.7–5.5)	4.0 (2.1–7.9)
hs-cTnI (Ortho)	0.3 (0.0–0.7)	0.6 (0.2–1.2)	0.9 (0.3–2.1)
Hs-cTn concentration > 99th URL (%)^a^			
hs-cTnT	2.4 (0.00)	5.8 (0.00)	15.6 (0.01)
hs-cTnI (Abbott)	0.5 (0.00)	1.0 (0.00)	2.4 (0.00)
hs-cTnI (Siemens)	0.8 (0.00)	1.9 (0.00)	3.4 (0.00)
hs-cTnI (Ortho)	0.8 (0.00)	1.5 (0.00)	3.7 (0.00)
Hs-cTn concentration > LoD (%)^a^			
hs-cTnT	81.3 (0.01)	93.9 (0.01)	97.0 (0.01)
hs-cTnI (Abbott)	41.3 (0.01)	65.5 (0.02)	74.4 (0.02)
hs-cTnI (Siemens)	60.0 (0.02)	76.9 (0.02)	81.8 (0.02)
hs-cTnI (Ortho)	39.9 (0.01)	59.3 (0.02)	70.3 (0.02)

^a^Concentrations of cardiac troponin were expressed as weighted median (interquartile range), and categorized variables were expressed as weighted proportions (standard error)

*pts* participants, *hs-cTn* high-sensitivity cardiac troponin, *URL* upper reference limit, *LoD* lower limit of detection

The derived 99th percentile of hs-cTnT from the presumably healthy diabetic population was higher than that of the healthy population and no overlap in the 95% CIs was found [15 (14–17) vs. 23 (18–30) ng/L]. In contrast, 99th percentiles (95% CI) of hs-cTnI (Abbott) in normoglycemic [13 (10–19) ng/L], prediabetic [11 (10–51) ng/L], and diabetic [7 (6–19) ng/L] populations were close and extensively overlapped. Hs-cTnI (Siemens) and hs-cTnI (Ortho) were similar to hs-cTnI (Abbott) regarding distributions and 99th URLs (Table 1, and Additional file 4).

### Prognostic value across glycemic status

Among 9714 participants, the median follow-up time was 16.8 years and 2633 died during follow-up (698 cardia-specific). The incidence rates of all-cause and cardiac-specific death per 1000 person-years were 7.29 (95% CI 6.71–7.88) and 1.60 (95% CI 1.31–1.89) for the normoglycemic population, 18.32 (95% CI 16.69–19.96) and 4.70 (95% CI 3.98–5.41) for the prediabetic population, and 38.00 (95% CI 34.68–41.32) and 11.92 (95% CI 9.94–13.89) for diabetic patients, respectively.

In both univariable and multivariable models, hs-cTnT and hs-cTnI (Abbott) were significantly associated with all-cause and cardiac-specific mortality across glycemic status (Table 2). In fully adjusted model 2, the hazard ratio per 1-standard deviation increase of natural log-transformed concentrations was 1.56 (95% CI 1.42–1.71; *P* < 0.001) for hs-cTnT and 1.35 (95% CI 1.17–1.55;* P* < 0.001) for hs-cTnI (Abbott) regarding all-cause mortality in the diabetic population.

**Table 2:** HR and 95%CIs of hs-cTnT and hs-cTnI (Abbott) concentrations regarding all-cause and cardiac-specific mortality

	Normoglycemia		Prediabetes		Diabetes	
	HR (95% CI)^a^	P-value	HR (95% CI)^a^	P-value	HR (95% CI)^a^	P-value
**All-cause mortality**						
Univariable						
hs-cTnT	3.03 (2.67–3.44)	< 0.001	2.91 (2.48–3.41)	< 0.001	1.97 (1.75–2.21)	< 0.001
hs-cTnI (Abbott)	3.09 (2.63–3.63)	< 0.001	3.15 (2.74–3.61)	< 0.001	2.23 (1.99–2.52)	< 0.001
hs-cTnI (Siemens)	2.75 (2.18–3.46)	< 0.001	3.73 (2.87–4.85)	< 0.001	2.33 (1.82–2.99)	< 0.001
hs-cTnI (Ortho)	3.79 (3.03–4.73)	< 0.001	4.18 (3.17–5.50)	< 0.001	2.79 (2.30–3.40)	< 0.001
Multivariable^b^						
hs-cTnT	1.31 (1.12–1.53)	< 0.001	1.46 (1.22–1.75)	< 0.001	1.56 (1.42–1.71)	< 0.001
hs-cTnI (Abbott)	1.36 (1.15–1.62)	< 0.001	1.65 (1.43–1.91)	< 0.001	1.35 (1.17–1.55)	< 0.001
hs-cTnI (Siemens)	1.06 (0.9–1.25)	0.48	1.55 (1.19–2.02)	0.001	1.21 (0.95–1.56)	0.13
hs-cTnI (Ortho)	1.31 (1.11–1.56)	0.002	1.36 (1.11–1.66)	0.003	1.24 (1.04–1.49)	0.02
**Cardiac-specific mortality**						
Univariable						
hs-cTnT	3.53 (3.05–4.08)	< 0.001	3.65 (2.75–4.84)	< 0.001	2.13 (1.80–2.51)	< 0.001
hs-cTnI (Abbott)	3.84 (3.07–4.82)	< 0.001	4.20 (3.46–5.10)	< 0.001	2.75 (2.21–3.41)	< 0.001
hs-cTnI (Siemens)	4.83 (3.08–7.56)	< 0.001	6.86 (5.06–9.3)	< 0.001	3.24 (1.95–5.38)	< 0.001
hs-cTnI (Ortho)	6.94 (4.65–10.4)	< 0.001	9.28 (5.81–14.8)	< 0.001	5.51 (3.70–8.21)	< 0.001
Multivariable^b^						
hs-cTnT	1.52 (1.23–1.86)	< 0.001	1.97 (1.37–2.83)	< 0.001	1.77 (1.48–2.12)	< 0.001
hs-cTnI (Abbott)	1.74 (1.34–2.26)	< 0.001	2.53 (1.87–3.44)	< 0.001	1.83 (1.33–2.53)	< 0.001
hs-cTnI (Siemens)	1.5 (0.91–2.47)	0.11	3.57 (2.15–5.95)	< 0.001	1.68 (0.84–3.34)	0.14
hs-cTnI (Ortho)	2.36 (1.41–3.95)	0.001	2.80 (1.34–5.83)	0.006	2.85 (1.76–4.62)	< 0.001

^a^Per 1-standard deviation increase of natural log-transformed hs-cTn concentrations

^b^Hs-cTn were adjusted in model 2 which incorporated age, sex, race-ethnicity, smoking status, systolic blood pressure, taking prescribed medication for hypertension, total cholesterol, and high-density lipoprotein cholesterol, estimated glomerular filtration rate, and N-terminal pro-brain natriuretic peptide

*HR* hazard ratio, *CI* confidence interval, *hs-cTn* high-sensitivity cardiac troponin

Like hs-cTnI (Abbott), there were significant associations between hs-cTnI (Ortho) and all-cause and cardiac-specific mortality across glycemic status (Table 2). However, hs-cTnI (Siemens) was significantly associated with all-cause and cardiac-specific mortality in the prediabetic population only.

In the primary-prevention population excluding patients with previous CVD, independent prognostic values of hs-cTnT and hs-cTnI (Abbott) remained across glycemic status (Additional file 5). However, hs-cTnI (Ortho) was associated with all-cause mortality in the normoglycemic population and with cardiac-specific mortality in the diabetic population. Hs-cTnI (Simens) was independently associated with cardiac-specific mortality in prediabetic and diabetic populations but had no association with all-cause mortality after adjustment.

### Incremental predictive value

In the normoglycemic population, the rPCE model has demonstrated a very good discriminative ability for 10-year cardiac-specific mortality (AUC = 0.889, 95% CI 0.863 to 0.916). Adding neither hs-cTnT nor hs-cTnI would improve the model performance (Table 3); however, adding hs-cTnI in company with hs-cTnT to the rPCE model significantly improved the discrimination (difference in AUC: 0.019; 95% CI 0.002 to 0.035,* P* = 0.02).

**Table 3:** The time-dependent AUC of predicting models regarding 10 year cardiac-specific mortality

	AUC	ΔAUC^a^	P-value^a^
**Normoglycemia (n = 5946)**			
rPCE	0.889 (0.863 to 0.916)	Ref	Ref
rPCE + hs-cTnT	0.905 (0.881 to 0.928)	0.015 (− 0.001 to 0.031)	0.06
rPCE + hs-cTnI	0.901 (0.876 to 0.926)	0.012 (− 0.004 to 0.028)	0.15
rPCE + hs-cTnT + hs-cTnI	0.908 (0.885 to 0.931)	0.019 (0.002 to 0.035)	0.02
**Prediabetes (n = 2172)**			
rPCE	0.810 (0.779 to 0.841)	Ref	Ref
rPCE + hs-cTnT	0.857 (0.824 to 0.891)	0.047 (0.018 to 0.076)	0.001
rPCE + hs-cTnI	0.851 (0.817 to 0.885)	0.041 (0.018 to 0.064)	< 0.001
rPCE + hs-cTnT + hs-cTnI	0.860 (0.827 to 0.894)	0.050 (0.021 to 0.079)	< 0.001
**Diabetes (n = 1596)**			
rPCE	0.706 (0.667 to 0.745)	Ref	Ref
rPCE + hs-cTnT	0.768 (0.734 to 0.803)	0.062 (0.038 to 0.086)	< 0.001
rPCE + hs-cTnI	0.777 (0.744 to 0.811)	0.071 (0.046 to 0.097)	< 0.001
rPCE + hs-cTnT + hs-cTnI	0.786 (0.753 to 0.818)	0.080 (0.053 to 0.107)	< 0.001

^a^The Difference in AUC between the rPCE model and the model plus either hs-cTnT or hs-cTnI or both

*AUC* area under the receiver operating characteristic curve, *rPCE* recalibrated Pooled Cohort Equation, *hs-cTn* high-sensitivity cardiac troponin

In the diabetic population, the predictive performance of the rPCE model was unsatisfactory (AUC = 0.706, 95% CI 0.667 to 0.745), adding either hs-cTnT or hs-cTnI or both could contribute to large amounts of increment in the discriminative ability of the rPCE model (Table 3). Moreover, the predictive performance of the rPCE risk score combined with hs-cTnT would be further improved by incorporating hs-cTnI (rPCE + hs-cTnT vs. rPCE + hs-cTnT + hs-cTnI, difference in AUC: 0.018; 95% CI 0.006 to 0.029; *P* = 0.002). However, the additive predictive value of hs-cTnI to hs-cTnT was not found in the normoglycemic population (difference in AUC: 0.003; 95% CI − 0.002 to 0.008; *P* = 0.20).

Prediabetes behaved like an intermediate status between normoglycemia and diabetes, among whom the rPCE model demonstrated a compromised predictive performance, and adding either hs-cTnT or hs-cTnI to the rPCE model would improve 10-year cardiac-specific mortality predictions (Table 3) but the additive value of hs-cTnI to hs-cTnT was also not found (difference in AUC: 0.003; 95% CI − 0.003 to 0.009; *P* = 0.37).

In the primary-prevention population, incremental predictive values of hs-cTnT and hs-cTnI for diabetes were consistent and robust (Additional file 6). In contrast, the combination of hs-cTnT and hs-cTnI could not improve the rPCE model performance in the normoglycemic population anymore. In the prediabetic population, hs-cTnI but not hs-cTnT had an incremental predictive value.

### Correlation between hs-cTnT and hs-cTnI

Similarly, correlation analyses of hs-cTnI focused on hs-cTnI (Abbott). We found a graded increase in correlation coefficients between hs-cTnT and hs-cTnI (Abbott) from normoglycemia (r = 0.38, 95% CI 0.36–0.40; *P* < 0.001), and prediabetes (r = 0.46, 95% CI 0.42–0.49; *P* < 0.001), to diabetes (r = 0.56, 95% CI 0.52–0.60; *P* < 0.001).

**Fig. 1:**
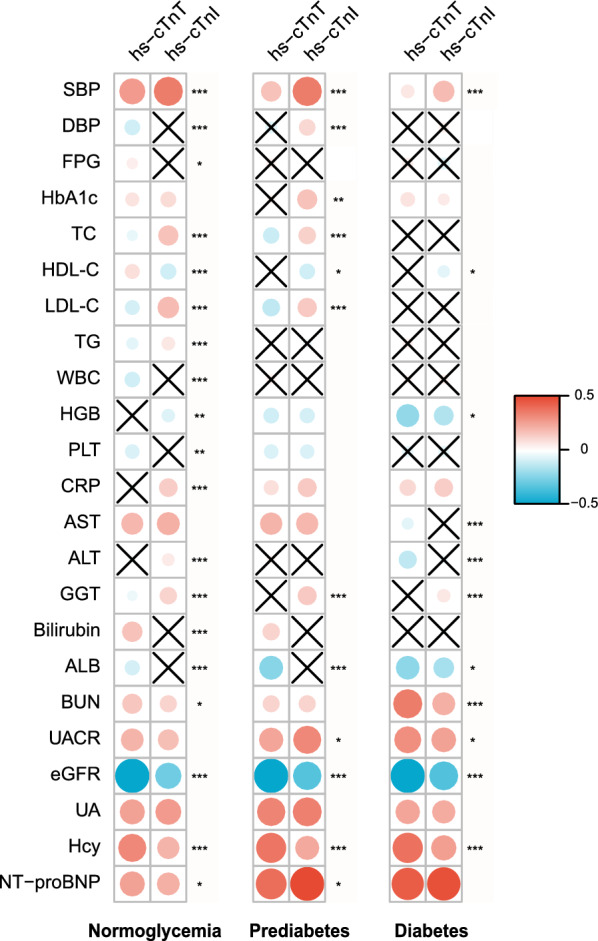
Correlations of clinical biomarkers with hs-cTnT and hs-cTnI across glycemic status. “** × **” in the grey box refers to non-significant correlations of the biomarker with hs-cTn. “*” at the right side of the boxes refers to a significant difference in the magnitude of correlation of the biomarker with hs-cTnT versus with hs-cTnI (Abbott); *, *P* < 0.05; **, *P* < 0.01; ***,* P* < 0.001. *SBP* systolic blood pressure, *DBP* diastolic blood pressure, *FPG* fasting plasma glucose, *TC* total cholesterol, *HDL-C* high-density lipoprotein cholesterol, *LDL-C* low-density lipoprotein cholesterol, *TG* triglyceride, WBC white blood cell count, *HGB* hemoglobin, *PLT* platelet count, *CRP* C-reactive protein, *AST* aspartate aminotransferase, *ALT* alanine transaminase, *GGT* gamma-glutamyl transferase, *ALB* serum albumin, *BUN* blood urea nitrogen, *UACR* urinary albumin creatinine ratio, *eGFR* estimated glomerular filtration rate, *UA* uric acid, *Hcy* homocysteine, *NT-proBNP* N-terminal pro-brain natriuretic peptide

The correlation pattern of hs-cTnT with biomarkers of renal function, inflammation, lipid metabolism, etc. across glycemic status was much different from hs-cTnI (Fig. 1 and Additional file 7). For instance, both assays were inversely correlated with eGFR but stronger for hs-cTnT irrespective of glycemic status. Hs-cTnI and hs-cTnT were both positively correlated with CRP concentrations in hyperglycemic populations with no difference in the magnitude of coefficients. In contrast, in the normoglycemic population, only hs-cTnI was significantly correlated with CRP, and the difference in coefficients between hs-cTnI and hs-cTnT was significant.

## Discussion

In the present study, we investigated and compared distributions, 99th percentiles, and prognostic and predictive values of hs-cTnT and hs-cTnI across glycemic status. We reported several findings with clinical implications for applications of hs-cTn in the context of hyperglycemia. First, concentrations of both hs-cTnI and hs-cTnT were higher in the diabetic population but the 99th percentile of hs-cTnT rather than hs-cTnI was susceptible to glycemic status. Next, both hs-cTnT and hs-cTnI provided independent prognostic values for all-cause and cardiac-specific mortality across glycemic status, but the additive value of hs-cTnI (Abbott) to hs-cTnT in predicting cardiac-specific mortality was robust in the diabetic but not the normoglycemic population. Finally, the correlation between hs-cTnT and hs-cTnI was modest in the normoglycemic population but was strengthened by hyperglycemia, moreover, a different pattern in correlations of CVD risk factors with hs-cTnT than hs-cTnI was found. These findings emphasized the critical interaction between hs-cTn and hyperglycemia.

Hyperglycemia was associated with increased levels of serum hs-cTn [22–24]. But whether hyperglycemia had an independent impact on URLs of hs-cTn is still under debate, moreover, rarely had a study directly compared the impact of hyperglycemia between hs-cTnT and hs-cTnI [24]. In the present study, presumably healthy diabetic and prediabetic participants with no underlying medical condition were selected by a stringent criterion same as for the healthy population except the glycemic requirement. Consequently, a larger number of subjects with subclinical diseases potentially affecting the heart were excluded so the pure impact of hyperglycemia on hs-cTn was unveiled [9, 15, 20, 24–26]. We found the derived 99th URL of hs-cTnT for diabetes was slightly higher than the manufacturer-proposed value (19 ng/L) as well as the value derived from the healthy normoglycemic population. In contrast, 99th percentiles of hs-cTnI irrespective of assays were very close across glycemic status and 95% CIs extensively overlapped. Similarly, Bluro et al. found the 99th percentile of hs-cTnT (Roche) derived from type 2 diabetes patients with no cardiovascular, renal, inflammatory, or systemic disease was over threefold of the manufacturer-proposed value (48 vs. 14 ng/L) [27]. Whereas, Krintus et al. found excluding individuals with HbA1c ≥ 6.5% from the presumably healthy population only had a marginal effect on the URL of Abbott hs-cTnI (11.1 vs. 11.2 ng/L, before and after exclusion) [28]. These cases indicated that the URL of hs-cTnT might be much more susceptible to glycemic status than hs-cTnI.

This large population-based study from NHANES, similar to prior studies based on the Multi-Ethnic Study of Atherosclerosis and the Atherosclerosis Risk In Communities study, indicated that hs-cTnT and hs-cTnI provided independent prognostic information on all-cause and cardiac-specific mortality consistently across glycemic status [24–26]. In certain clinical conditions, incorporating emerging cardiac biomarkers (e.g., hs-cTn, NT-proBNP) into classical CVD risk scores may contribute to a substantial improvement [9]. As risk stratification plays an important role in informing treatment for diabetes and prediabetes, introducing hs-cTn would help to identify high-risk diabetes and prediabetes who may benefit from intensive anti-diabetic treatments [18].

Nevertheless, unlimitedly accumulating biomarkers in the predictive model may improve the performance in a not cost-effective way and increase the model complexity. An appropriate approach is incorporating biomarkers that represent distinct pathophysiology. Therefore, with prior knowledge of the excellent correlation between hs-cTnI and hs-cTnT in acute myocardial infarction, there is a very small number of studies investigating the predictive utility of the combination of hs-cTnT and hs-cTnI and such studies across glycemic status were even scarcer, even though the independent association between increased hs-cTn and worse CVD outcomes was well-recognized [24–26, 29–32]. However, we found simultaneously measuring hs-cTnT and hs-cTnI may provide non-redundant information on CVD risk stratification compared to a single measurement. The non-interchangeable predictive value of hs-cTnI with hs-cTnT in the diabetic but not in the normoglycemic population indicated hs-cTnI and hs-cTnT in the context of chronic hyperglycemia possibly represented distinct pathophysiological processes, which supported the differential association of hyperglycemia with hs-cTnT and hs-cTnI [6–8, 32–36].

Possible mechanisms of differential associations of hyperglycemia with cTnT and cTnI were poorly discovered. Clearance of cTn was primarily dependent on the kidney but the molecular weight of cTnT was higher than cTnI [10]. Therefore, we observed strong correlations of both hs-cTnT and hs-cTnI with eGFR but hs-cTnT was more strongly correlated. UACR is another critical biomarker of renal function associated with diabetic nephropathy [37]. Hs-cTnT was also found to be more strongly correlated with UACR than hs-cTnI in the diabetic but not in the normoglycemic population. These findings suggested the stronger association of hs-cTnT with diabetes than hs-cTnI might be partially attributed to discordant clearance rates between cTnT and cTnI in diabetic participants with known or unknown renal impairment [5–7, 10].

Furthermore, skin autofluorescence, a biomarker of advanced glycation end-products, was associated with both serum hs-cTnT concentrations and sarcopenia in diabetic patients [38, 39]. In patients with skeletal muscle disease, transcriptions of genes coding for cTnT were up-regulated in skeletal muscles but that of cTnI was at normal level [8]. Ectopic releases of cTnT from damaged skeletal muscle by advanced glycation end-products could also be a potential source for discordance but this needs more research.

Interestingly, the age- and sex-adjusted correlation between hs-cTnT and hs-cTnI was modest in the normoglycemic population but was more pronounced in prediabetic and diabetic populations. We presumed there might be different but also shared pathways of hyperglycemia-mediated cTnT and cTnI elevations that strengthen their correlation. Indeed, hs-cTnI and hs-cTnT were comparably correlated with CRP in hyperglycemic populations while in the normoglycemic population hs-cTnI but not hs-cTnT was correlated with CRP. As inflammation played an important role in the development of diabetic cardiomyopathy [40], this finding suggested inflammation might serve as a common pathway that concordantly increased concentrations of hs-cTnT and hs-cTnI in hyperglycemic populations.

This study has some limitations. First, as an observational study, the association should never be arbitrarily treated as a causal effect. More research is needed to illustrate the effect of hyperglycemia on the myocardium and cTn from various aspects. Second, the number of presumably healthy diabetic participants was relatively small due to the stringent criteria, as recommended by IFCC at least 400 healthy males and females are needed to obtain the precise 99th percentile for clinical use [20]. However, this study aimed to investigate the influence of glycemic status on URLs of hs-cTn rather than to derive ready-to-use values for diagnosing myocardial infarction. Third, NHANES is designed to be representative of the civilian, non-institutionalized population in the US, however, socioeconomic, ethnic, epidemiological, nutritional, and genetic characteristics of whom may be significantly different from populations outside the US, therefore, the conclusion from this study may not be generalized to populations outside the US. Fourth, the time interval between blood draws and hs-cTn measurements may also influence serum concentrations of hs-cTn [41]. Fifth, there are various assays to measure serum hs-cTnI concentrations so our conclusions may not be extended to other assays. Finally, large population-based studies were warranted to further evaluate the cost-effectiveness of single vs. dual-assay measurement before introducing this strategy to clinical practice.

## Conclusions

This population-based study demonstrated consistent prognostic associations of hs-cTnT and hs-cTnI across glycemic status but robust incremental predictive values of hs-cTnI and hs-cTnT were found in the diabetic population only. Moreover, the susceptibility of hs-cTnT 99th percentiles to diabetes than hs-cTnI, the non-interchangeable role of hs-cTnI with hs-cTnT in diabetic CVD risk stratification, and the different pattern in correlations with CVD risk factors between hs-cTnT and hs-cTnI across glycemic status suggested elevated hs-cTnT and hs-cTnI may be associated with different pathological processes in the context of chronic hyperglycemia. However, more research is required to further illustrate the interaction between hyperglycemia and elevations of hs-cTn.

### Supplementary Information

**Additional file 1: **Laboratory assays to determine biomarker concentrations.

**Additional file 2: Table S1.** Demographic and clinical characteristics of the population with full availability of four hs-cTn assays stratified by glycemic status.

**Additional file 3: Figure S1.** Volin plot of serum hs-cTnT (Roche), hs-cTnI (Abbott), hs-cTnI (Siemens), and hs-cTnI (Ortho) concentrations.

**Additional file 4: Table S2. **99th percentile and 95% CI of hs-cTn across glycemic status.

**Additional file 5: Table S3. **HRs and 95%CIs per 1-standard deviation increase of natural log-transformed concentrations of hs-cTnT and hs-cTnI regarding all-cause and cardiac-specific mortality in the primary-prevention population excluding patients with previous CVD.

**Additional file 6: Table S4. **The time-dependent AUC of the rPCE model predicting 10-year cardiac-specific mortality vs. the rPCE model plus either hs-cTnT or hs-cTnI or both in the primary-prevention population excluding patients with previous CVD.

**Additional file 7: Table S5. **Correlation coefficients (%) of clinical biomarkers with hs-cTnT and hs-cTnI across glycemic status.

## Data Availability

The datasets analyzed during the current study are available in the NHANES database (https://wwwn.cdc.gov/nchs/nhanes/default.aspx.)

## References

[1] Byrne RA, Rossello X, Coughlan JJ, Barbato E, Berry C, Chieffo A, Claeys MJ, Dan GA, Dweck MR, Galbraith M (2023). 2023 ESC Guidelines for the management of acute coronary syndromes. Eur Heart J.

[2] Jia X, Sun W, Hoogeveen RC, Nambi V, Matsushita K, Folsom AR, Heiss G, Couper DJ, Solomon SD, Boerwinkle E (2019). High-sensitivity troponin I and incident coronary events, stroke, heart failure hospitalization, and mortality in the ARIC study. Circulation.

[3] McEvoy JW, Daya N, Tang O, Fang M, Ndumele CE, Coresh J, Christenson RH, Selvin E (2023). High-sensitivity troponins and mortality in the general population. Eur Heart J.

[4] Rubini Gimenez M, Twerenbold R, Reichlin T, Wildi K, Haaf P, Schaefer M, Zellweger C, Moehring B, Stallone F, Sou SM (2014). Direct comparison of high-sensitivity-cardiac troponin I vs. T for the early diagnosis of acute myocardial infarction. Eur Heart J.

[5] Sattar N, Mills NL, Porteous D, Campbell A, Woodward M, Welsh C, Padmanabhan S, Hayward C, McConnachie A, Boachie C (2018). Comparison between high-sensitivity cardiac troponin T and cardiac troponin I in a large general population cohort. Clin Chem.

[6] Kimenai DM, Martens RJH, Kooman JP, Stehouwer CDA, Tan FES, Schaper NC, Dagnelie PC, Schram MT, van der Kallen CJH, Sep SJS (2017). Troponin I and T in relation to cardiac injury detected with electrocardiography in a population-based cohort—the maastricht study. Sci Rep.

[7] Omland T, Pfeffer MA, Solomon SD, de Lemos JA, Rosjo H, Saltyte Benth J, Maggioni A, Domanski MJ, Rouleau JL, Sabatine MS (2013). Prognostic value of cardiac troponin I measured with a highly sensitive assay in patients with stable coronary artery disease. J Am Coll Cardiol.

[8] de Fay Lavallaz J, Prepoudis A, Wendebourg MJ, Kesenheimer E, Kyburz D, Daikeler T, Haaf P, Wanschitz J, Loscher WN, Schreiner B (2022). Skeletal muscle disorders: a noncardiac source of cardiac troponin T. Circulation.

[9] Tang O, Matsushita K, Coresh J, Ndumele C, McEvoy JW, Sharrett AR, Hoogeveen R, Ballantyne CM, Selvin E (2020). High-sensitivity cardiac troponin I and T for cardiovascular risk stratification in adults with diabetes. Diabetes Care.

[10] Hijazi Z, Siegbahn A, Andersson U, Lindahl B, Granger CB, Alexander JH, Atar D, Gersh BJ, Hanna M, Harjola VP (2015). Comparison of cardiac troponins I and T measured with high-sensitivity methods for evaluation of prognosis in atrial fibrillation: an ARISTOTLE substudy. Clin Chem.

[11] Zelniker TA, Wiviott SD, Mosenzon O, Goodrich EL, Jarolim P, Cahn A, Bhatt DL, Leiter LA, McGuire DK, Wilding J (2023). Association of cardiac biomarkers with major adverse cardiovascular events in high-risk patients with diabetes: a secondary analysis of the DECLARE-TIMI 58 trial. JAMA Cardiol.

[12] Januzzi JL, Butler J, Jarolim P, Sattar N, Vijapurkar U, Desai M, Davies MJ (2017). Effects of canagliflozin on cardiovascular biomarkers in older adults with type 2 diabetes. J Am Coll Cardiol.

[13] Centers for Disease Control and Prevention/National Center for Health Statistics. Questionnaires, Datasets, and Related Documentation for NHANES 1999–2000. https://wwwn.cdc.gov/nchs/nhanes/continuousnhanes/default.aspx?BeginYear=1999. Accessed July 10, 2023.

[14] The International Federation of Clinical Chemistry Committee on Clinical Application of Cardiac Bio-Markers. High-Sensitivity Cardiac Troponin I and T Assay Analytical Characteristics Designated by Manufacturer v092021. 2022. https://ifcc.web.insd.dk/media/479205/highsensitivity-cardiac-troponin-i-and-t-assay-analyticalcharacteristics-designated-by-manufacturerv092021-3.pdf. Accessed July 10, 2023.

[15] McEvoy JW, Tang O, Wang D, Ndumele CE, Coresh J, Christenson RH, Selvin E (2023). Myocardial injury thresholds for 4 high-sensitivity troponin assays in U.S. adults. J Am Coll Cardiol.

[16] Centers for Disease Control and Prevention/National Center for Health Statistics. High-sensitivity cardiac troponins (Surplus) (SSTROP_A). https://wwwn.cdc.gov/Nchs/Nhanes/1999-2000/SSTROP_A.htm. Accessed July 10, 2023.

[17] Centers for Disease Control and Prevention. National Diabetes Statistics Report website. https://www.cdc.gov/diabetes/data/statistics-report/index.html. Accessed July 09, 2023.

[18] ElSayed NA, Aleppo G, Aroda VR, Bannuru RR, Brown FM, Bruemmer D, Collins BS, Hilliard ME, Isaacs D, Johnson EL (2023). Classification and diagnosis of diabetes: standards of care in diabetes-2023. Diabetes Care.

[19] Inker LA, Eneanya ND, Coresh J, Tighiouart H, Wang D, Sang Y, Crews DC, Doria A, Estrella MM, Froissart M (2021). New creatinine- and cystatin C-based equations to estimate GFR without race. N Engl J Med.

[20] Aakre KM, Saenger AK, Body R, Collinson P, Hammarsten O, Jaffe AS, Kavsak P, Omland T, Ordonez-Lianos J, Apple FS (2022). Analytical considerations in deriving 99th percentile upper reference limits for high-sensitivity cardiac troponin assays: educational recommendations from the IFCC committee on clinical application of cardiac bio-markers. Clin Chem.

[21] Yadlowsky S, Hayward RA, Sussman JB, McClelland RL, Min YI, Basu S (2018). Clinical implications of revised pooled cohort equations for estimating atherosclerotic cardiovascular disease risk. Ann Intern Med.

[22] Rubin J, Matsushita K, Ballantyne CM, Hoogeveen R, Coresh J, Selvin E (2012). Chronic hyperglycemia and subclinical myocardial injury. J Am Coll Cardiol.

[23] Myhre PL, Lyngbakken MN, Berge T, Roysland R, Aagaard EN, Pervez O, Kvisvik B, Brynildsen J, Norseth J, Tveit A (2021). Diagnostic thresholds for pre-diabetes mellitus and diabetes mellitus and subclinical cardiac disease in the general population: data from the ACE 1950 study. J Am Heart Assoc.

[24] Selvin E, Lazo M, Chen Y, Shen L, Rubin J, McEvoy JW, Hoogeveen RC, Sharrett AR, Ballantyne CM, Coresh J (2014). Diabetes mellitus, prediabetes, and incidence of subclinical myocardial damage. Circulation.

[25] Nguyen K, Fan W, Bertoni A, Budoff MJ, Defilippi C, Lombardo D, Maisel A, Szklo M, Wong ND (2020). N-terminal pro B-type natriuretic peptide and high-sensitivity cardiac troponin as markers for heart failure and cardiovascular disease risks according to glucose status (from the multi-ethnic study of atherosclerosis [MESA]). Am J Cardiol.

[26] Tang O, Daya N, Matsushita K, Coresh J, Sharrett AR, Hoogeveen R, Jia X, Windham BG, Ballantyne C, Selvin E (2020). Performance of high-sensitivity cardiac troponin assays to reflect comorbidity burden and improve mortality risk stratification in older adults with diabetes. Diabetes Care.

[27] Bluro IM, Nardi MA, De Miguel R, Fernández M, Rolando JY, Abraham Fóscolo MM, Denaday LR, Candenas N, Cagide AM, Pizarro R (2021). Distribution and prognostic value of high-sensitive troponin T measurement in patients with type 2 diabetes without cardiovascular disease. Endocrinol Diabetes Nutr.

[28] Krintus M, Kozinski M, Boudry P, Lackner K, Lefevre G, Lennartz L, Lotz J, Manysiak S, Shih J, Skadberg O (2015). Defining normality in a European multinational cohort: critical factors influencing the 99th percentile upper reference limit for high sensitivity cardiac troponin I. Int J Cardiol.

[29] Scirica BM, Bhatt DL, Braunwald E, Raz I, Cavender MA, Im K, Mosenzon O, Udell JA, Hirshberg B, Pollack PS (2016). Prognostic implications of biomarker assessments in patients with type 2 diabetes at high cardiovascular risk: a secondary analysis of a randomized clinical trial. JAMA Cardiol.

[30] Witkowski M, Wu Y, Hazen SL, Tang WHW (2021). Prognostic value of subclinical myocardial necrosis using high-sensitivity cardiac troponin T in patients with prediabetes. Cardiovasc Diabetol.

[31] Sabbatinelli J, Giuliani A, Bonfigli AR, Ramini D, Matacchione G, Campolucci C, Ceka A, Tortato E, Rippo MR, Procopio AD (2022). Prognostic value of soluble ST2, high-sensitivity cardiac troponin, and NT-proBNP in type 2 diabetes: a 15-year retrospective study. Cardiovasc Diabetol.

[32] Echouffo-Tcheugui JB, Musani SK, Bertoni AG, Correa A, Fox ER, Mentz RJ (2022). Patients phenotypes and cardiovascular risk in type 2 diabetes: the jackson heart study. Cardiovasc Diabetol.

[33] Welsh P, Woodward M, Hillis GS, Li Q, Marre M, Williams B, Poulter N, Ryan L, Harrap S, Patel A (2014). Do cardiac biomarkers NT-proBNP and hsTnT predict microvascular events in patients with type 2 diabetes? Results from the ADVANCE trial. Diabetes Care.

[34] Jende JME, Groener JB, Kender Z, Hahn A, Morgenstern J, Heiland S, Nawroth PP, Bendszus M, Kopf S, Kurz FT (2020). Troponin T parallels structural nerve damage in type 2 diabetes: a cross-sectional study using magnetic resonance neurography. Diabetes.

[35] Bojer AS, Sorensen MH, Vejlstrup N, Goetze JP, Gaede P, Madsen PL (2020). Distinct non-ischemic myocardial late gadolinium enhancement lesions in patients with type 2 diabetes. Cardiovasc Diabetol.

[36] Hicks CW, Wang D, McDermott K, Matsushita K, Tang O, Echouffo-Tcheugui JB, McEvoy JW, Christenson RH, Selvin E (2023). Associations of cardiac biomarkers with peripheral artery disease and peripheral neuropathy in US adults without prevalent cardiovascular disease. Arterioscler Thromb Vasc Biol.

[37] Navaneethan SD, Zoungas S, Caramori ML, Chan JCN, Heerspink HJL, Hurst C, Liew A, Michos ED, Olowu WA, Sadusky T (2023). Diabetes management in chronic kidney disease: synopsis of the KDIGO 2022 clinical practice guideline update. Ann Intern Med.

[38] Waqas K, Chen J, Trajanoska K, Ikram MA, Uitterlinden AG, Rivadeneira F, Zillikens MC (2022). Skin autofluorescence, a noninvasive biomarker for advanced glycation end-products, is associated with sarcopenia. J Clin Endocrinol Metab.

[39] Yoshioka K (2018). Skin autofluorescence is associated with high-sensitive cardiac troponin T, a circulating cardiac biomarker, in Japanese patients with diabetes: a cross-sectional study. Diab Vasc Dis Res.

[40] Sanganalmath SK, Dubey S, Veeranki S, Narisetty K, Krishnamurthy P (2023). The interplay of inflammation, exosomes and Ca(2+) dynamics in diabetic cardiomyopathy. Cardiovasc Diabetol.

[41] Giannitsis E, Katus HA (2017). Concerns about the stability of hsTnI assay after 20 years of storage. J Am Coll Cardiol.

