# Supplementary material for: Distribution and prognostic value of high-sensitivity cardiac troponin T and I across glycemic status: a population-based study
Source: Cardiovasc Diabetol. 2024 Feb 24;23:83. doi: 10.1186/s12933-023-02092-z (PMC10894468; doi:10.1186/s12933-023-02092-z)
Supplement: Supplementary file 1 — Additional file 1: Laboratory assays to determine biomarker concentrations. [file 12933_2023_2092_MOESM1_ESM.docx]

**Laboratory assays to determine biomarker concentrations**

Very low concentrations of high-sensitivity cardiac troponin (hs-cTn) were initially blinded in clinical routine due to greater variability near the lower limits. In NHANES, however, such values were unblinded for research purposes. If unblinding was not feasible, values were imputed as the lower limits of detection divided by the square root of 2 for hs-cTn T (Roche) and hs-cTn I (Ortho) while the hs-cTnI (Siemens) value was imputed as 2.5 ng/L (the limit of quantitation divided by the square root of 2). The hs-cTnI (Abbott) was not blinded for research purposes so values even below the lower limits of detection were reported as initial.

N-terminal pro-brain natriuretic peptide (NT-proBNP) was measured in serum on the Roche Cobas e601 autoanalyzer with the lower and upper limits of detection being 5 pg/ml and 35000 pg/ml, respectively. Serum cystatin C concentration was measured using a Cystatin C immunoassay on Siemens Dimension Vista 1500 analyzer (Siemens Healthcare Diagnostics), the lower and upper limits of detection of which are 0.23 mg/L and 8.00 mg/L, respectively. C-reactive protein was measured by latex-enhanced nephelometry on a Behring Nephelometer.

HbA1c in NHANES 1999–2004 cycles were measured as a prespecified part of the protocols. Whole blood samples were shipped to the Diabetes Diagnostic Laboratory at the University of Missouri-Columbia, and then HbA1c was determined on a fully automated glycohemoglobin analyzer (Primus CLC330 and Primus CLC 385) utilizing the principle of boronate affinity high-performance liquid chromatography.

A detailed description of the laboratory method used to measure alanine transaminase, aspartate aminotransferase, albumin, gamma-glutamyl transferase, bilirubin, creatinine, blood urea nitrogen, glucose, cholesterol, triglycerides, uric acid, homocysteine, hemoglobin, platelet, and white blood cell count can be found at NHANES website. (https://wwwn.cdc.gov/nchs/nhanes/continuousnhanes/labmethods.aspx?BeginYear=2001)
