# Supplementary material for: Distribution and prognostic value of high-sensitivity cardiac troponin T and I across glycemic status: a population-based study
Source: Cardiovasc Diabetol. 2024 Feb 24;23:83. doi: 10.1186/s12933-023-02092-z (PMC10894468; doi:10.1186/s12933-023-02092-z)
Supplement: Supplementary file 2 — Additional file 2: Table S1. Demographic and clinical characteristics of the population with full availability of four hs-cTn assays stratified by glycemic status. [file 12933_2023_2092_MOESM2_ESM.docx]

**eTable 1. Demographic and clinical characteristics of the population with full availability of four hs-cTn assays stratified by glycemic status**

| Variable ^a^ | Normoglycemia | Prediabetes | Diabetes |
| --- | --- | --- | --- |
| No. of pts before weighting | 5946 | 2172 | 1596 |
| Age, yrs | 40.1 (0.4) | 52.7 (0.4) | 58.7 (0.6) |
| Male | 44.1 (0.01) | 55.3 (0.01) | 51.5 (0.01) |
| Race/ethnicity |  |  |  |
| non-Hispanic White | 70.4 (0.02) | 70.5 (0.02) | 64.4 (0.03) |
| Mexican American | 7.8 (0.01) | 7.3 (0.01) | 7.5 (0.01) |
| non-Hispanic Black | 11.1 (0.01) | 11.5 (0.01) | 14.8 (0.02) |
| other Hispanic | 6.1 (0.01) | 5.9 (0.01) | 6.0 (0.02) |
| other | 4.6 (0.00) | 4.9 (0.01) | 7.3 (0.01) |
| BMI |  |  |  |
| normal | 89.5 (0.01) | 81.6 (0.01) | 70.9 (0.02) |
| obesity | 9.0 (0.01) | 17.7 (0.01) | 28.8 (0.02) |
| underweight | 1.5 (0.00) | 0.7 (0.00) | 0.2 (0.00) |
| Current smoker | 27.0 (0.01) | 21.7 (0.01) | 20.0 (0.01) |
| Prior cardiovascular disease | 4.8 (0.00) | 10.6 (0.01) | 25.8 (0.02) |
| Hypertension | 20.5 (0.01) | 44.1 (0.01) | 65.4 (0.01) |
| SBP, mmHg | 119 (0.4) | 129 (0.5) | 132 (0.7) |
| eGFR<60ml/min/1.73m^2^ | 1.9 (0.00) | 4.8 (0.00) | 12.6 (0.01) |
| Albuminuria | 5.9 (0.00) | 11.9 (0.01) | 29.7 (0.01) |

^a^ Continuous and categorized variables were expressed as weighted mean (SE) and weighted proportions (SE), respectively; concentrations of cardiac troponin were expressed as weighted median (IQR). Abbreviation: BMI, body mass index; SBP, systolic blood pressure; eGFR, estimated glomerular filtration rate.
