# Supplementary material for: Distribution and prognostic value of high-sensitivity cardiac troponin T and I across glycemic status: a population-based study
Source: Cardiovasc Diabetol. 2024 Feb 24;23:83. doi: 10.1186/s12933-023-02092-z (PMC10894468; doi:10.1186/s12933-023-02092-z)
Supplement: Supplementary file 3 — Additional file 3: Figure S1. Volin plot of serum hs-cTnT (Roche), hs-cTnI (Abbott), hs-cTnI (Siemens), and hs-cTnI (Ortho) concentrations. [file 12933_2023_2092_MOESM3_ESM.pdf]

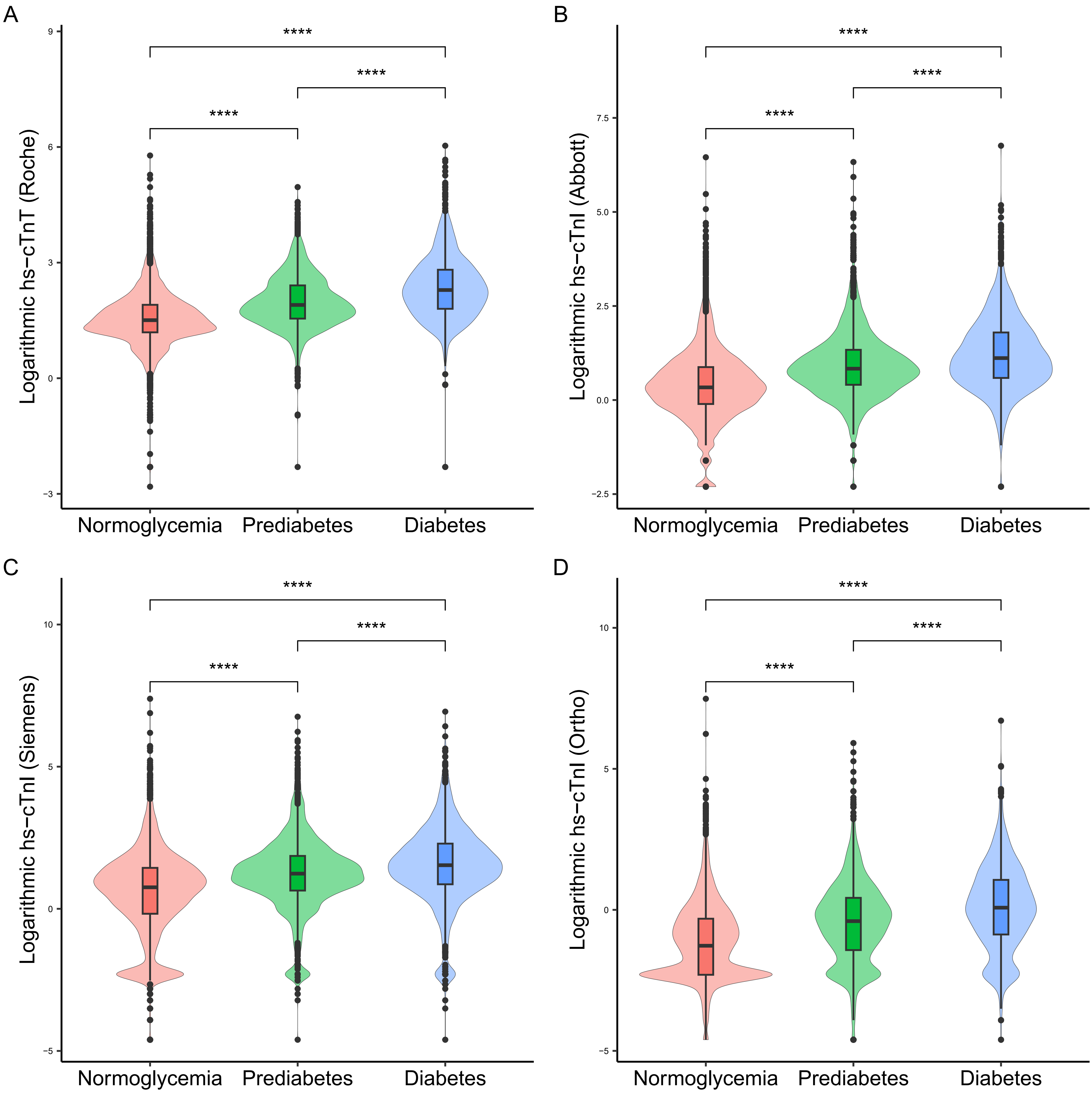

eFigure 1. Volin plot of serum hs-cTnT (Roche) (A), hs-cTnI (Abbott) (B), hs-cTnI (Siemens) (C), and hs-cTnI (Ortho) (D) concentrations. Between-group differences were evaluated by Wilcoxon rank sum test, **** refers to P-value < 0.0001. hs-cTn: high-sensitivity cardiac troponin.
