# Supplementary material for: Distribution and prognostic value of high-sensitivity cardiac troponin T and I across glycemic status: a population-based study
Source: Cardiovasc Diabetol. 2024 Feb 24;23:83. doi: 10.1186/s12933-023-02092-z (PMC10894468; doi:10.1186/s12933-023-02092-z)
Supplement: Supplementary file 4 — Additional file 4: Table S2. 99th percentile and 95% CI of hs-cTn across glycemic status. [file 12933_2023_2092_MOESM4_ESM.docx]

**eTable 2. 99^th^ percentile and 95%CI of hs-cTn across glycemic status.**

|  | Normoglycemia | | Prediabetes | | Diabetes | |
| --- | --- | --- | --- | --- | --- | --- |
|  | N | 99^th^ percentile ^a^ | N | 99^th^ percentile | N | 99^th^ percentile |
| **99th percentile (95%CI)** | | | | | | |
| hs-cTnT (Roche) | 1786 | 15 (14 to 17) | 482 | 18 (16 to 28) | 126 | 23 (18 to 30) |
| hs-cTnI (Abbott) | 1796 | 13 (10 to 19) | 476 | 11 (10 to 51) | 126 | 7 (6 to 19) |
| hs-cTnI (Siemens) | 1749 | 36 (27 to 74) | 457 | 38 (25 to 45) | 125 | 22 (11 to 47) |
| hs-cTnI (Ortho) | 1786 | 4 (3 to 8) | 478 | 4 (3 to 9) | 126 | 4 (3 to 5) |

^a^ The unit is ng/L.

Abbreviation: CI, confidence interval; hs-cTn, high-sensitivity cardiac troponin.
