# Supplementary material for: Distribution and prognostic value of high-sensitivity cardiac troponin T and I across glycemic status: a population-based study
Source: Cardiovasc Diabetol. 2024 Feb 24;23:83. doi: 10.1186/s12933-023-02092-z (PMC10894468; doi:10.1186/s12933-023-02092-z)
Supplement: Supplementary file 5 — Additional file 5: Table S3. HRs and 95%CIs per 1-standard deviation increase of natural log-transformed concentrations of hs-cTnT and hs-cTnI regarding all-cause and cardiac-specific mortality in the primary-prevention population excluding patients with previous CVD. [file 12933_2023_2092_MOESM5_ESM.docx]

**eTable 3. HRs and 95%CIs per 1-standard deviation increase of natural log-transformed concentrations of hs-cTnT and hs-cTnI regarding all-cause and cardiac-specific mortality in the primary-prevention population excluding patients with previous CVDs.**

|  | Normoglycemia | | Prediabetes | | Diabetes | |
| --- | --- | --- | --- | --- | --- | --- |
|  | HR (95%CI) | P-value | HR (95%CI) | P-value | HR (95%CI) | P-value |
| **All-cause mortality** ^a^ | | | | | | |
| hs-cTnT | 1.28 (1.08-1.52) | 0.005 | 1.35 (1.08-1.69) | 0.009 | 1.47 (1.29-1.68) | <0.001 |
| hs-cTnI (Abbott) | 1.25 (1.04-1.50) | 0.02 | 1.54 (1.28-1.86) | <0.001 | 1.25 (1.04-1.50) | 0.02 |
| hs-cTnI (Siemens) | 0.98 (0.83-1.16) | 0.84 | 1.31 (0.96-1.79) | 0.09 | 1.15 (0.91-1.47) | 0.24 |
| hs-cTnI (Ortho) | 1.20 (1.01-1.43) | 0.04 | 1.20 (0.98-1.46) | 0.07 | 1.00 (0.82-1.22) | 0.99 |
| **Cardiac-specific mortality** ^a^ | | | | | | |
| hs-cTnT | 1.36 (1.03-1.8) | 0.03 | 1.61 (1.05-2.49) | 0.03 | 1.58 (1.25-2.00) | <0.001 |
| hs-cTnI (Abbott) | 1.51 (1.07- 2.13) | 0.02 | 2.24 (1.51-3.32) | <0.001 | 1.59 (1.25-2.02) | <0.001 |
| hs-cTnI (Siemens) | 1.22 (0.61- 2.45) | 0.57 | 2.56 (1.37-4.79) | 0.003 | 1.89 (1.41-2.54) | <0.001 |
| hs-cTnI (Ortho) | 1.73 (0.87- 3.45) | 0.12 | 2.00 (0.82-4.92) | 0.13 | 2.02 (1.28-3.19) | 0.003 |

^a^ Hs-cTnI (Siemens) and hs-cTnI (Ortho) were adjusted in model 2 which incorporated age, sex, race-ethnicity, smoking status, systolic blood pressure, taking prescribed medication for hypertension, total cholesterol, and high-density lipoprotein cholesterol, estimated glomerular filtration rate, and N-terminal pro-brain natriuretic peptide.

Abbreviations: HR, hazard ratio; CI, confidence interval; hs-cTn, high-sensitivity cardiac troponin.
