# Supplementary material for: Distribution and prognostic value of high-sensitivity cardiac troponin T and I across glycemic status: a population-based study
Source: Cardiovasc Diabetol. 2024 Feb 24;23:83. doi: 10.1186/s12933-023-02092-z (PMC10894468; doi:10.1186/s12933-023-02092-z)
Supplement: Supplementary file 6 — Additional file 6: Table S4. The time-dependent AUC of the rPCE model predicting 10-year cardiac-specific mortality vs. the rPCE model plus either hs-cTnT or hs-cTnI or both in the primary-prevention population excluding patients with previous CVD. [file 12933_2023_2092_MOESM6_ESM.docx]

**eTable 4. The time-dependent AUC of the rPCE model predicting10-year CVD mortality vs. the rPCE model plus either hs-cTnT or hs-cTnI or both in the primary-prevention population excluding patients with previous CVD.**

|  | AUC ^a^ | ΔAUC ^a^ | P-value |
| --- | --- | --- | --- |
| **Normoglycemia (n=5615)** |  |  |  |
| rPCE | 0.877 (0.838 to 0.916) | ref. | ref. |
| rPCE + hs-cTnT | 0.885 (0.847 to 0.922) | 0.008 (-0.013 to 0.029) | 0.46 |
| rPCE + hs-cTnI | 0.878 (0.837 to 0.918) | 0.001 (-0.021 to 0.022) | 0.96 |
| rPCE + hs-cTnT + hs-cTnI | 0.887 (0.85 to 0.923) | 0.009 (-0.012 to 0.031) | 0.38 |
| **Prediabetes (n=1899)** |  |  |  |
| rPCE | 0.830 (0.791 to 0.87) | ref. | ref. |
| rPCE + hs-cTnT | 0.857 (0.814 to 0.9) | 0.027 (-0.006 to 0.059) | 0.10 |
| rPCE + hs-cTnI | 0.854 (0.811 to 0.896) | 0.023 (0.001 to 0.045) | 0.04 |
| rPCE + hs-cTnT + hs-cTnI | 0.861 (0.818 to 0.904) | 0.031 (-0.001 to 0.062) | 0.05 |
| **Diabetes (n=1161)** |  |  |  |
| rPCE | 0.751(0.697 to 0.805) | ref. | ref. |
| rPCE + hs-cTnT | 0.789 (0.738 to 0.839) | 0.038 (0.014 to 0.061) | 0.002 |
| rPCE + hs-cTnI | 0.793 (0.746 to 0.839) | 0.041 (0.019 to 0.063) | <0.001 |
| rPCE + hs-cTnT + hs-cTnI | 0.798 (0.75 to 0.846) | 0.047 (0.022 to 0.072) | <0.001 |

^a^ The Difference in AUC between the rPCE model and the model plus either hs-cTnT or hs-cTnI or both.

Abbreviations: AUC, area under the receiver operating characteristic curve; rPCE, recalibrated Pooled Cohort Equation; hs-cTn, high-sensitivity cardiac troponin.
