# Supplementary material for: Distribution and prognostic value of high-sensitivity cardiac troponin T and I across glycemic status: a population-based study
Source: Cardiovasc Diabetol. 2024 Feb 24;23:83. doi: 10.1186/s12933-023-02092-z (PMC10894468; doi:10.1186/s12933-023-02092-z)
Supplement: Supplementary file 7 — Additional file 7: Table S5. Correlation coefficients (%) of clinical biomarkers with hs-cTnT and hs-cTnI across glycemic status. [file 12933_2023_2092_MOESM7_ESM.docx]

**eTable 5. Correlation coefficients (%) of clinical biomarkers with hs-cTnT and hs-cTnI across glycemic status.**

|  | Normoglycemia ^a^ |  |  | Prediabetes ^a^ |  |  | Diabetes ^a^ |  |  |
| --- | --- | --- | --- | --- | --- | --- | --- | --- | --- |
|  | hs-cTnT | hs-cTnI | P ^b^ | hs-cTnT | hs-cTnI | P ^b^ | hs-cTnT | hs-cTnI | P ^b^ |
| SBP | **19.0 (16.5 to 21.4)** | **24.1 (21.6 to 26.5)** | <0.001 | **9.3 (5.2 to 13.4)** | **20.0 (16.0 to 24.0)** | <0.001 | **6.8 (2.0 to 11.7)** | **18.5 (13.7 to 23.3)** | <0.001 |
| DBP | **-6.4 (-8.9 to -3.9)** | 1.1 (-1.4 to 3.6) | <0.001 | -2.8 (-6.9 to 1.3) | **5.9 (1.8 to 10.1)** | <0.001 | -3.1 (-7.9 to 1.8) | 3.9 (-0.9 to 8.8) | NA |
| PFG | **3.1 (0.6 to 5.6)** | -0.1 (-2.6 to 2.4) | 0.02 | 1.9 (-2.2 to 6.0) | -3.5 (-7.6 to 0.6) | NA | 4.3 (-1.7 to 10.3) | -5.2 (-11.2 to 0.8) | NA |
| HbA1c | **4.9 (2.4 to 7.4)** | **6.4 (3.9 to 8.9)** | 0.30 | 2.1 (-2 to 6.2) | **9.0 (4.9 to 13.1)** | 0.002 | **8.0 (3.2 to 12.9)** | **5.0 (0.1 to 9.8)** | 0.18 |
| TC | **-2.9 (-5.4 to -0.4)** | **11.1 (8.6 to 13.6)** | <0.001 | -5.7 (-9.8 to -1.5) | **6.7 (2.6 to 10.8)** | <0.001 | -1.7 (-6.6 to 3.1) | 1 (-3.8 to 5.9) | NA |
| HDL-C | **5.7 (3.2 to 8.2)** | **-6.7 (-9.2 to -4.2)** | <0.001 | 0.3 (-3.8 to 4.5) | **-5.2 (-9.3 to -1.0)** | 0.01 | 0.1 (-4.7 to 5) | **-5.5 (-10.3 to -0.7)** | 0.01 |
| LDL-C | **-5.6 (-8.8 to -2.4)** | **12.8 (9.6 to 16.0)** | <0.001 | **-6.8 (-11.6 to -2.1)** | **8.2 (3.4 to 12.9)** | <0.001 | -6.4 (-14.1 to 1.3) | 2.8 (-4.9 to 10.5) | NA |
| TG | **-3.6 (-6.1 to -1.1)** | **4.7 (2.2 to 7.2)** | <0.001 | -3.1 (-7.2 to 1.0) | -1.5 (-5.6 to 2.7) | NA | 3.8 (-1.1 to 8.6) | 1.6 (-3.2 to 6.5) | NA |
| WBC | **-6.2 (-8.7 to -3.7)** | 0.5 (-2.0 to 3.1) | <0.001 | 1.9 (-2.2 to 6.1) | 3.7 (-0.5 to 7.8) | NA | 2.6 (-2.3 to 7.4) | 4.4 (-0.5 to 9.2) | NA |
| HGB | -0.3 (-2.8 to 2.2) | **-4.5 (-7.0 to -2.0)** | 0.003 | **-5.2 (-9.3 to -1.1)** | **-4.9 (-9.0 to -0.8)** | 0.89 | **-21.4 (-26.1 to -16.6)** | **-15.6 (-20.4 to -10.8)** | 0.01 |
| PLT | **-5.4 (-7.9 to -2.9)** | -1.0 (-3.5 to 1.5) | 0.002 | **-4.4 (-8.5 to -0.2)** | **-4.3 (-8.4 to -0.1)** | 0.96 | -4.3 (-9.1 to 0.5) | -4.3 (-9.2 to 0.5) | NA |
| CRP | -1.2 (-3.7 to 1.3) | **9.3 (6.8 to 11.8)** | <0.001 | **4.7 (0.6 to 8.9)** | **7.9 (3.8 to 12.1)** | 0.14 | **10.4 (5.6 to 15.2)** | **13.6 (8.8 to 18.4)** | 0.15 |
| AST | **13.3 (10.8 to 15.8)** | **14.6 (12.2 to 17.1)** | 0.33 | **11.2 (7.1 to 15.3)** | **11.1 (7.0 to 15.2)** | 0.98 | **-5.1 (-10.0 to -0.3)** | 3.2 (-1.6 to 8.1) | <0.001 |
| ALT | -1.2 (-3.7 to 1.3) | **4.0 (1.4 to 6.5)** | <0.001 | 1.6 (-2.5 to 5.8) | 3.8 (-0.4 to 7.9) | NA | **-12.8 (-17.6 to -8.0)** | -2.4 (-7.3 to 2.4) | <0.001 |
| GGT | **-2.6 (-5.2 to -0.1)** | **8.0 (5.5 to 10.5)** | <0.001 | -0.3 (-4.4 to 3.8) | **7.9 (3.8 to 12.0)** | <0.001 | -1.0 (-5.8 to 3.9) | **6.6 (1.8 to 11.5)** | <0.001 |
| Bilirubin | **11.3 (8.8 to 13.8)** | 0.6 (-1.9 to 3.1) | <0.001 | **6.5 (2.4 to 10.6)** | 2.9 (-1.2 to 7) | 0.10 | 0.3 (-4.5 to 5.2) | -1.8 (-6.6 to 3.1) | NA |
| ALB | **-5.9 (-8.4 to -3.4)** | -1.2 (-3.7 to 1.4) | <0.001 | **-13 (-17.1 to -8.9)** | -3.2 (-7.3 to 1.0) | <0.001 | **-22.8 (-27.5 to -18.1)** | **-17.2 (-22.0 to -12.4)** | 0.01 |
| BUN | **10.9 (8.4 to 13.4)** | **7.7 (5.2 to 10.2)** | 0.03 | **6.2 (2.0 to 10.3)** | **6.3 (2.2 to 10.4)** | 0.94 | **35.4 (30.8 to 39.9)** | **21.3 (16.5 to 26.0)** | <0.001 |
| UACR | **14.3 (11.8 to 16.8)** | **12 (9.5 to 14.5)** | 0.10 | **13.4 (9.3 to 17.5)** | **18.2 (14.1 to 22.2)** | 0.03 | **30.0 (25.4 to 34.6)** | **25.0 (20.3 to 29.7)** | 0.02 |
| eGFR | **-34.1 (-36.5 to -31.7)** | **-19.3 (-21.8 to -16.8)** | <0.001 | **-27.9 (-31.9 to -23.9)** | **-18.8 (-22.8 to -14.7)** | <0.001 | **-50.0 (-54.2 to -45.8)** | **-34.8 (-39.4 to -30.3)** | <0.001 |
| UA | **17.6 (15.2 to 20.1)** | **18.8 (16.4 to 21.3)** | 0.4001 | **18.6 (14.6 to 22.7)** | **19.5 (15.4 to 23.5)** | 0.69 | **24.4 (19.6 to 29.1)** | **22.0 (17.2 to 26.7)** | 0.28 |
| Hcy | **22.4 (20.0 to 24.9)** | **13.7 (11.2 to 16.2)** | <0.001 | **21.1 (17.1 to 25.1)** | **13.1 (9.0 to 17.1)** | <0.001 | **38.4 (33.9 to 42.9)** | **26.2 (21.5 to 30.9)** | <0.001 |
| NT-proBNP | **17.5 (15.0 to 19.9)** | **14.4 (11.9 to 16.9)** | 0.03 | **22.3 (18.3 to 26.3)** | **27.4 (23.4 to 31.4)** | 0.02 | **44.1 (39.8 to 48.5)** | **47.4 (43.1 to 51.6)** | 0.10 |

^a^ Coefficients were expressed as percentages (%), The bold value refer to a significant correlation.

^b^ Comparisons between hs-cTnT and hs-cTnI in the magnitude of correlation coefficients. If neither of the two assays was significantly correlated with the biomarker, the P value would not be calculated and NA would be given.

Abbreviation: SBP, systolic blood pressure; DBP, diastolic blood pressure; PFG, plasma fasting glucose; TC, total cholesterol; HDL, high-density lipoprotein cholesterol; LDL, low-density lipoprotein cholesterol TG, triglyceride; WBC, white blood cell count; HGB, hemoglobin; PLT, platelet count; CRP, C-reactive protein; AST, aspartate aminotransferase; ALT, alanine transaminase; GGT, gamma-glutamyl transferase; ALB, serum albumin; BUN, blood urea nitrogen; UACR, urinary albumin creatinine ratio; eGFR, estimated glomerular filtration rate; UA, uric acid; Hcy, homocysteine; NT-proBNP, N-terminal pro-brain natriuretic peptide.
